# Modelling of road traffic fatalities in India

**DOI:** 10.1016/j.aap.2017.12.019

**Published:** 2018-03

**Authors:** Rahul Goel

**Affiliations:** MRC Epidemiology Unit, University of Cambridge, UK

**Keywords:** India, Traffic fatalities, Distance-decay functions, Accident prediction model, Ecological model

## Abstract

Passenger modes in India include walking, cycling, buses, trains, intermediate public transport modes (IPT) such as three-wheeled auto rickshaws or tuk-tuks, motorised two-wheelers (2W) as well as cars. However, epidemiological studies of traffic crashes in India have been limited in their approach to account for the exposure of these road users. In 2011, for the first time, census in India reported travel distance and mode of travel for workers. A Poisson-lognormal mixture regression model is developed at the state level to explore the relationship of road deaths of all the road users with commute travel distance by different on-road modes. The model controlled for diesel consumption (proxy for freight traffic), length of national highways, proportion of population in urban areas, and built-up population density. The results show that walking, cycling and, interestingly, IPT are associated with lower risk of road deaths, while 2W, car and bus are associated with higher risk. Promotion of IPT has twofold benefits of increasing safety as well as providing a sustainable mode of transport. The mode shift scenarios show that, for similar mode shift across the states, the resulting trends in road deaths are highly dependent on the baseline mode shares. The most worrying trend is the steep growth of death burden resulting from mode shift of walking and cycling to 2W. While the paper illustrates a limited set of mode shift scenarios involving two modes at a time, the model can be applied to assess safety impacts resulting from a more complex set of scenarios.

## Highlights

• First study in India to account for exposure of six different modes of transport for injury modelling.
• Auto rickshaws or tuk-tuks are found to be the only motorised mode associated with higher safety.
• Increase in walking and cycling is associated with higher safety.
• Inverse U-shaped pattern of road deaths results from a mode shift from 2W to cars.

## 1 Introduction

India has one of the highest shares of road traffic fatalities in the world. A large proportion of these fatalities are pedestrians, cyclists, and riders of motorised two-wheelers (2W) (Hsiao et al., 2013; Mohan et al., 2015). This is because a large share of daily trips is contributed by the three modes. According to Census 2011 in India (Census-India, 2017a), the three modes contribute up to 70% of work trips. India lacks government-led efforts for transport-related data in terms of travel surveys or traffic counts. As a result, road traffic injury models been limited in their approach to account for exposure of multiple road user groups. At most, models have used vehicle registration numbers of 2W and cars which highly overestimate actual in-use fleet (Goel et al., 2015; 2016). Moreover, registration data does not account for walking, cycling and use of public transport (PT) and is therefore limited in its application.

Current levels of vehicle ownership in India are far lower than most high-income countries. In 2011, only 6% of all the households in India owned a car, compared to more than 75% households in many high-income countries (Census-India, 2017b; Statista, 2017). As a result, a large proportion of population continues to walk, cycle, or use PT. At the same time private vehicle ownership witnesses an inevitable growth. From 1990 to 2015, the average year-on-year growth rate of 2W and cars was 9–10%, implying that private motorised fleet is doubling every 7–8 years. This rate is many times higher than the growth rate of population and, therefore, indicates a dramatic mode shift from walking, cycling and PT to private vehicle use.

Motorisation in India is also different from many of the high-income countries in two main aspects. Firstly, motorised traffic in India is dominated by 2W. For every car in India, there are more than five times as many 2W (MoRTH, 2013). In case of a crash, ceteris paribus, a 2W rider is many times more vulnerable to an injury than a car driver. Thus, a motorisation based on 2W makes its road users more risk-prone. A car-based motorisation, on the other hand, ensures higher safety of vehicle occupants. Secondly, PT modes in India include not only buses and trains but also a range of other intermediate modes such as auto rickshaws and tuk-tuks, common in many south-Asian settings. They serve the purpose of PT and, at the same time, have a smaller engine capacity than a bus or even a car. Thus, an impact of these modes on safety is important from the perspective of transport policies.

In summary, travel patterns in India present a complex and a unique mix of traffic modes and are going through rapid changes. All these changes are occurring in the context of poor enforcement of traffic laws, as well as a lack of safe infrastructure for walking and cycling. Given this background, travel patterns are likely to be a strong predictor of the number of road injuries. This is the first time Census in India has included travel-related information. The information includes the mode of travel and the travel distance for workers. This gives an opportunity to explore how the travel patterns are related to road deaths to assess and design travel demand and traffic safety policies in India.

## 2 Objectives

The main objective of this study is to develop an ecological model of road traffic fatalities, with states of India as areal units. The model aims to establish a relationship between total annual road fatalities and commute travel distance by different modes, while controlling for state-specific confounders. I aim to develop a model with a form often used in injury modelling and shown in Eq. (1):

(1) $$n={M1}^{e1}{M2}^{e2}M3^{e3}e^{\sum \beta_{i}x_{i}}$$

where, M1, M2, and M3 represent travel distance (or volume) of the three road user categories, e1, e2, and e3 represent their respective exponents, $x_{i}$ represents a set of predictor variables which control for factors other than volume, and $\beta_{i}$ their corresponding coefficients. The values of exponents and coefficients are obtained using regression modelling.

The three road user categories have been used only for illustration. This form of the model is achieved by anti-logging a log-linear relationship between injury counts (*n*) and the volume or distance variables (M1, M2, M3). It is a usual practice to include these variables in their logged form. This also results in multiplicative risk factors as shown in Eq. (1). Such models are often referred to as accident prediction models. This name, however, is an oxymoron, since accidents by nature are not predictable, and therefore a more scientific term should be injury prediction models.

The models have been developed at a range of level of aggregation. These includes traffic junctions, roundabouts, crossings, or road sections among the ‘micro ‘or ‘meso-level’ models to city wards, traffic zones, and municipalities among the ‘macro-level’ or ecological models (Elvik and Bjørnskau, 2017). For this paper, I will only discuss models at areal or macro levels. The outcome variable in these models also vary based on the objective of the study. Most models include number of injuries or crashes of a specific road user as outcome. In such models, the exposure variables include the volume of that road user (injuries of which are outcome variable) along with the volume of conflicting road user. For instance, models with pedestrian injuries as outcome and pedestrian and car volume as explanatory variables. No model in the literature has accounted for more than two road users, except Elvik (2016) who modelled pedestrian injuries using volume of cars, cyclists and pedestrians.

The model presented in this paper differs from the previous literature in two main aspects. First, the dependent variable in the model is the number of road deaths of all road users and not specific to a single road user. Second, the model accounts for multiple modes as explanatory variables, and not just two modes, thus reflecting the heterogeneity of traffic on Indian roads. Thus, the model in this paper aims to establish a relationship between overall road death burden and a mix of travel modes. This also implies that a comparison of the results presented in this paper with the literature needs to be done cautiously.

My aim to develop this model is twofold—analytical and for prediction. The former will be achieved my assessing the magnitude and signs of exponents of different road users. The latter will be achieved by simulating future travel patterns to assess their impact on road deaths. I will explain these using an example. Suppose that there are three road users in the model specified in Eq. (1), and from the regression modelling it is estimated that the two of them (say, M1 and M2) have positive exponents (e1 and e2) and one (say, M3) has a negative exponent (e3).

From an analytical perspective, this implies that an increase in M1 and M2 will increase injury burden, while an increase in M3 will reduce it. Among M1 and M2, the comparison between the magnitudes of their exponents will also illustrate which of the two modes will result in higher injury burden if both are increased by the same amount. From a prediction perspective, one can model what-if scenarios of mode shift and understand the trajectories of road death burden. For instance, mode shift from M3 (mode associated with less risk) to M1 or M2 (modes associated with higher risk) will result in much higher death burden than mode shift within M1 and M2.

The literature on accident prediction models is also divided among those where the exposure of different road users (such as M1, M2, and M3 in the example above) are in the form of counts (or volumes) and those where it is in the form of distance (Elvik and Bjørnskau, 2017; Schepers and Heinen, 2013**)**. When the units of analysis are point locations or of a consistent size, the counts can be justified as an exposure variable. For instance, counts of motor vehicles, pedestrians, or cyclists at traffic junctions, road sections, or traffic analysis zones in a city. If the units of analyses differ in their size, the counts may be an incomplete measure. The models with only counts also eliminate the possibility to predict changes in injuries if population travelled using the same modes however the distance of travel changed. Therefore, a model with distance is more robust in its application to predict changes in injuries resulting from changing travel patterns.

## 3 Data

The model explained in the previous section needs three main data types—a) annual number of road deaths for each state as dependent variable, b) mode-specific commute travel distance, and c) other explanatory variables. In 2011, India had 28 states and 7 Union Territories (UTs). The average population of the UTs is 2.9 million while that of the states is 41 million. Two of the UTs are islands, Andaman and Nicobar Island in the east and Lakshadweep in the west, and contribute 0.04% of the total population of the country. These were excluded from the analysis. The remaining 28 states and 5 UTs will be referred to as 33 states henceforth. Note that Delhi, the capital city of India, is a city-state and is therefore included as one of the units in this analysis. The states cover a large range of population from 0.24 million to 200 million. Table 1 presents the descriptive statistics.

**Table 1:** Descriptive statistics of variables.

	Mean	Median	Std. Deviation	Minimum	Maximum
Average annual number of fatalities (2010–2012)	4139	2221	4894	24	15669
Population	36,679,089	25,351,462	44,957,638	243,247	199,812,341
Average fatality rate (per 100,000 population)	11.6	12.1	4.9	2.3	22.4
Total commute distance by Walk (km)	2,817,833	1,874,778	3,060,533	39,947	11,049,986
Total commute distance by Cycle (km)	4,318,576	2,844,264	5,808,973	3738	26,810,567
Total commute distance by 2W (km)	6,064,611	3,876,805	7,491,930	13,554	28,539,315
Total commute distance by Car (km)	2,342,221	1,163,472	2,716,398	13,581	9,114,740
Total commute distance by IPT (km)	1,729,288	917,165	2,415,759	25,242	8,979,712
Total commute distance by Bus (km)	14,271,637	5,772,699	18,475,298	49,906	82,321,109
Total commute distance by Train (km)	9,597,981	2,468,720	18,804,973	8212	88,281,102
Annual Diesel Consumption (×1000 tonnes)	1957	930	2217	48	7483
Percent Urban Population	38%	30%	22%	10%	98%
Length of national highway (km)	2009	1512	1790	1	5874
Built-up Density (persons per km^2^)	12260	11062	6347	2582	26066

National Crime Records Bureau of India publishes annual number of road accidents, number of people injured, and number of deaths for each state and UT in India. Number of injury crashes are highly underestimated in India (Mohan et al., 2015), and as a result only number of deaths have been used for the analysis. Corresponding to census year, I used average number of fatalities for the three years (2010 through 2012) for stable estimates (NCRB, 2011; 2012; 2013).

**Fig. 1:**
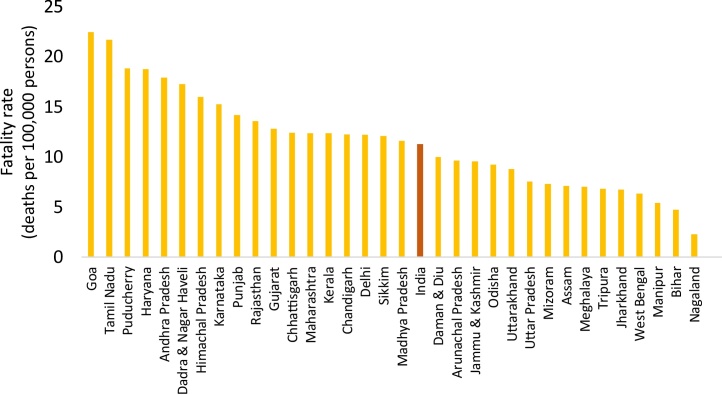
Average annual road fatality rates across states and all India.

In almost all the states, year-to-year variation of number of road deaths was minimal across the three years (see Appendix: Table A1). There are, however, two exceptions—Punjab, where number of road deaths corresponding to three years are 2133 (2010), 4897 (2011) and 4795 (2012), and Nagaland, with 71 (2010), 106 (2011), and 44 (2012) deaths. In both the states, highest number of deaths is more than 2 times higher than the lowest number. Average fatality rate across the states is 11.6 per 100,000 persons and vary from 2.3 to 22.4. Fig. 1 presents fatality rates for all the 33 states and overall India in a descending order.

In this analysis, I have excluded deaths occurring at railway crossings, which is an area where on-road modes and trains interact. Over the three years, total number of road deaths at railway crossing are 3344 (2010), 2366 (2011), and 1808 (2012). In contrast, total number of road deaths for the three years are 133938, 136834 and 139091 respectively (NCRB, 2011; 2012; 2013), thus, deaths on railway crossing is 1–3% of the on-road deaths.

In 2011, Census of India introduced two questions regarding the commute of workers (Census-India, 2017a). These questions were asked from a subset of all workers—the category called ‘other workers’. This category excludes those involved in agricultural or household-based activities. The category of ‘other workers’ represent 42% of all the workers in India (Census-India, 2017c). The two questions on commuting included mode of travel and one-way distance (in kilometres) from residence to place of work, and in the former only one mode could be selected. For further details see Goel, 2018.

The question on mode thus disregards the multimodal characteristics of some of the trips. However, census provides no details in this regard (Census-India, 2017d). Thus, the working assumption is that the respondents informed their main mode of travel—the one using which they covered the longest travel distance. Since the census is conducted using personal interviews, it is possible that these questions, in some cases, were answered by proxy respondents, for instance, by other members of the household. However, no such information is available from census to account for this bias.

There are 9 options for the travel modes: (1) walk, (2) cycle, (3) moped/scooter/motorcycle, (4) car, (5) tempo/auto rickshaw/taxi, (6) bus, (7) train, (8) water transport, and (9) any other, and an option of ‘No travel’. Category 3 is referred to as motorised two wheelers (2W), and category 5 as IPT. The latter consists of intermediate public transport, or para-transit modes such as three-wheeled auto rickshaws, common across India (for their description see Goel and Tiwari, 2016 and Kumar et al., 2016).

For each mode, Census has reported mode-specific count of workers classified into 7 distance categories: 0–1 km, 2–5 km, 6–10 km, 11–20 km, 21–30 km, 31–50 km, and >50 km. Walking has been reported up to 10 km, and cycling up to 30 km. The data has been reported only at the aggregate level of states and districts, with a further classification into rural, urban and total. In this analysis, total data (urban plus rural) has been used at state level. Also, water transport and ‘any other’ categories were excluded. These modes were reported by 1.2% of those travelling by one of the 9 travel modes. The detailed method to estimate average distance travelled by each mode in each state is presented in Goel (2018). I used average distance (see Appendix: Table A2) and multiplied by corresponding number of workers to estimate total commute distance travelled by each mode. Table 1 presents the descriptive statistics of the total distance.

One of the major limitations of distance estimates from census data is that it is limited to only commuting while our dependent variable includes road deaths from all travel. Therefore, I investigate the relationship between commute distance and the variables which represent overall travel. A study was commissioned by MoPNG to estimate sectoral share of petrol and diesel consumption in India. According to this, Petrol is only used by cars and 2W, except 2% by IPT (Nielsen, 2013). Thus, petrol consumption is a good indicator of total distance travelled by 2W and cars. The Pearson correlation of annual petrol consumption at state level (MoPNG, 2012) with commute distance estimated for 2W is 0.98 and with commute distance of car is 0.92. This indicates that commute distance by these two modes is a good proxy of overall travel distance by the two modes. Distance by car has less correlation than 2W since cars also use diesel and, to a lesser extent, compressed natural gas (MoPNG, 2012; Goel and Guttikunda, 2015).

In case of buses, I use distance travelled by public buses to compare. These buses are operated by government-run organisations known as State Road Transport Undertakings (SRTUs). There is a correlation of 0.89 between passenger kilometres reported by SRTU buses in the states (MoRTH, 2011) and the commute distance travelled by buses. The data for SRTU was available for 17 out of 33 states. Note that bus transport in India is carried out by public as well as private buses. Therefore, public buses do not represent all the buses, yet they represent bus travel for all purposes.

There is no data to see correlations for walk, cycle, IPT or trains. However, given the high correlations for other modes, it can be assumed that these correlations will be consistent across all the modes. Commute pattern as a proxy for overall travel has also been reported for England. Goodman (2013) reported strong linear relationships between mode shares from Census-reported commuting and those from national travel survey (which includes all trips) for multiple transport modes in England.

To control for state-specific factors other variables need to be included. While distance travelled by passenger transport modes are accounted for, a large proportion of road deaths are caused in crashes with goods vehicles (Goel, 2017; Mohan et al., 2016; Naqvi and Tiwari, 2017). According to a study commissioned by the Ministry of Petroleum and Natural Gas (MoPNG), on an average, 70% of the diesel in the country is consumed by road transport vehicles in India. Within road transport (70%), 22% is consumed by cars, 28% by trucks, 10% by buses, and 6% by three-wheeled passengers and goods vehicles (Nielsen, 2013). Taxis, included within cars, run mostly on diesel (Goel and Guttikunda, 2015). Thus, diesel consumption can be used as a proxy of vehicle kilometres travelled by freight as well as taxis. Annual diesel consumption for year 2011–12 was used from annual publication of MoPNG (MoPNG, 2012).

The other variables include proportion of population living in urban areas, population density and length of national highways. All the three variables can be categorised as built environment variables and are expected to interact with the exposure of road users in positive or negative direction. Urban areas have different traffic patterns than rural areas, population density have been reported to have a significant effect on traffic safety and length of national highway determines inter-state connectivity and amount of long-distance traffic.

Census reports population classified by urban and rural. I calculated the proportion of total population living in urban areas and refer to it as level of urbanisation. The level of urbanisation varies from 10% to 98% (Census-India, 2012). Population density of the states were calculated using state-level urban built-up area reported by the National Remote Sensing Centre through their web portal, Bhuvan **(**NRSC, 2016). The NRSC reports Land use Land Cover data using multi-temporal satellite data of 2011–12 from Resourcesat-2 LISS III images. We used the sum of urban and rural areas reported in NRSC data and expressed the density as persons per km^2^. Only national highways have been used since their reporting is likely to be more reliable and consistent as they are maintained by a single organisation across the country—National Highway Authority of India. The length of NHs reported for year 2011 were used (MoRTH, 2013).

## 4 Method

### 4.1 Cluster analysis

The regression model is aimed at understanding the independent effects of distance travelled by different modes. However, it is also worth understanding how travel patterns vary across the states, and how this variation relates to fatality rates. Within each state, I expressed total commute distance (estimated as described in previous section) of each mode as the proportion of total commute distance across all the modes. The proportions for each state total to unity and I refer to these as mode shares. Given a large variation in the magnitude of total distance travelled, mode shares enable comparison across the states.

Following this, I used k-means clustering to classify states into a group of clusters with similar distribution of mode share. In this method, states are assigned into clusters such that the sum of the squared deviations (Euclidian distance) from each observation and the cluster centroid is minimised. This sum is called within-cluster sum of squares (WSS). States were classified into 5 clusters, based on the optimum value of total WSS across all clusters. This means that an increment in the number of clusters beyond 5 does not further reduce the value of WSS.

### 4.2 Regression model

As this analysis is at the state level, it is constrained by a small number of observations (*n* = 33). In this context, use of Bayesian methods rather than frequentist method is preferred especially if various explanatory variables are included (Hox et al., 2012). I modelled fatalities with Poisson-lognormal mixture using Bayesian hierarchical modelling. The modelling was done using R-INLA (Rue et al., 2009) which is an R package and employs integrated nested Laplace approximations to estimate the posterior distributions. The package has been used for injury modelling by DiMaggio (2015) and Goel et al. (2018). The hierarchical model is described as follows:

(2) $$y_{n}=Poisson\left(f_{n}\right)$$

(3) $$\log\left(f_{n}\right)=\log\left(e_{n}\right)+\beta_{0}+\beta X_{n}+\delta_{n}$$

(4) $$\delta_{n}∼N(0,1/\tau_{\delta })$$

(5) $${log(\tau }_{\delta })∼logGamma(1,0.0005)$$

where, $y_{n}$are the observed annual fatality counts of all road users in state n, $f_{n}$ are the expected count of fatalities, $X_{n}$ represents a vector of explanatory variables, $e_{n}$ is the exposure, $\beta_{0}$ is the intercept, β is a vector of fixed effect parameters, and $\delta_{n}$ is the uncorrelated heterogeneity or unstructured error. Here, $\delta_{n}$ represents overdispersion and accounts for variation in the expected fatality risk after controlling for the independent variables. Population of each state represents the corresponding exposure ($e_{n})$.

The first level of the hierarchical modelling framework presented in the Eq.(2) through (5) is the likelihood model or the random sampling of number of fatalities ($y_{n})$from a Poisson distribution with a state-specific expected count ($f_{n}$). The second level models the log-linear relationship between expected fatality risk and independent variables. The logged relationship ensures positive values of $f_{n}.$Note that exposure ($e_{i}$) is an offset (a covariate with coefficient value 1) and, therefore, effectively acts as a denominator for left-hand side of the equation and expresses it as population risk (log ($\lambda_{n})$ = $log(f_{n}/e_{n})$). Therefore, this modelling framework accounts for exposed population explicitly, rather than treating it as a covariate.

The prior for $\delta_{n}$ is modelled as normal distribution where $\tau_{\delta }$ refers to the precision of the distribution and is inverse of the variance. Note that from Eq. (3), $f_{n}$ can be expressed as $e^{\beta_{0}}.e^{\beta X_{n}}.e^{\delta_{n}}$, therefore, in effect, the log of the overdispersion term ($e^{\delta_{n}}$) has a normal distribution, thus the distribution of $e^{\delta_{n}}$is lognormal. This makes the model a Poisson-lognormal mixture model. Further, the hyper parameter, ${log(\tau }_{\delta }$) is assigned a prior of log-gamma distribution with shape and inverse-scale parameters of 1 and 0.0005, respectively. Using log of $\tau_{\delta }$ ensures a positive value as it represents standard deviation. These priors with large standard deviations assume no prior knowledge of the effects and are referred to as uninformative priors.

Census only reports main mode of travel, therefore, in case of PT, this will result in underestimation of walking to and from the PT stops. To account for this, I assumed 1 km of walking distance corresponding to each trip of a PT mode (IPT, bus and train) longer than 1 km. The sensitivity of this assumption on the model results was tested by assuming no walking distance and assuming 1.5 km as the walking distance. While train distance is not included in the model, its walking distance is included as this distance is covered on the roads. Note that 1 km includes walking on either side of a PT trip, and is likely a conservative estimate for many trips which involve much longer than 500 m to walk to PT stops.

Four regression models are presented varying from minimally controlled to maximally controlled. The additional explanatory variables are added into the models in three stages. **Model 1** (minimally controlled) includes only commute distance variables, **model 2** adds diesel consumption, **model 3** has additional adjustment of length of National Highways (**model 3**), and **model 4** also includes population density and percent urban population. Model 1 accounts only for passenger travel modes, model 2 also accounts for freight modes, and models 3 and 4 account for built environment and related features.

The estimates of Bayesian modelling are in the form of posterior distributions of all parameters—coefficients as well as error terms. The results of these distributions will be presented in the form of mean and standard deviation, after ensuring that the distributions are normally distributed in which case mean is a suitable central tendency. Their significance will be reported based on whether zero lies within 90% or 95% Bayesian Confidence intervals (BCI). A coefficient significant at 95% BCI, for example, means that the range which covers 95% of all the values of a posterior distribution does not include zero.

## 5 Results

### 5.1 Cluster analysis

**Fig. 2:**
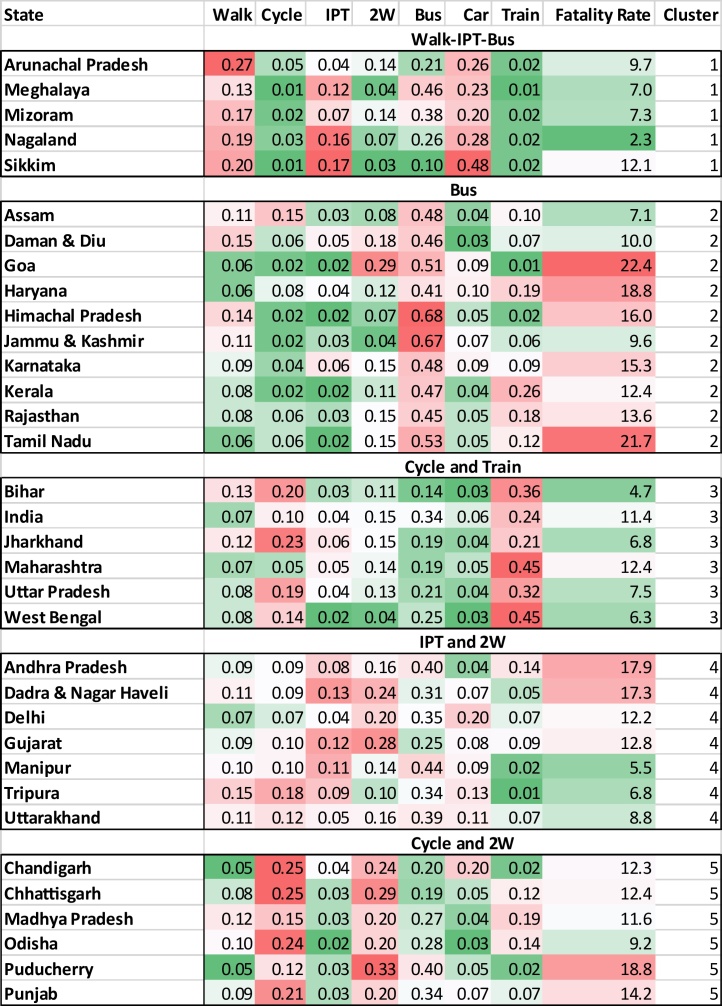
Mode shares within each state and corresponding cluster. (Shades of green represents below average values, white represent average, and shades of red represent above average; darker colours represent values farther from average; titles for each cluster represent the group of modes at above average levels in each cluster).

The result of cluster analysis is presented in Fig. 2 which also includes all India. The figure includes mode shares, fatality rates and the cluster number. The cells for mode shares and fatality rates are highlighted with colour scales. The shades of red represent values greater than the average across all the states, white represents average values, and shades of green represent values lower than average. This can also be used to identify modes that define a cluster. I titled each cluster with a group of modes that the cluster comprises at their above-average levels represented with shades of red.

Table 2 presents average mode shares within each cluster. Within each cluster, the mode with a high share is also highlighted, same as those in the cluster titles in Fig. 2. Cluster 1 consists of states with high levels of walk, IPT and car, cluster 2 consists of states with high level of bus use, cluster 3 consists of high levels of cycle and train, cluster 4 has high levels of 2W and IPT, and cluster 5 has high levels of cycle and 2W. Cluster 2 is the only one with a high share of bus use.

**Table 2:** Average mode share by cluster (Shaded cells represent modes with high share in the cluster).

The appearance of the same colour band for a given mode and fatality rate represents a positive correlation between the two. For instance, in Cluster 1, the shades of green for 2W, cycle, and train appear with shades of green of fatality rates, thus indicating a positive correlation between each of the three modes and fatality risk. In cluster 3, the shades of green for bus and car appear with shares of green of fatality rates, thus indicating a positive correlation of the two modes with the fatality risk. Median fatality rate for the 5 clusters are—7.7, 14.7, 8.2, 12.3, and 13.1 fatalities per 100,000 persons for clusters 1 through 5, respectively.

It is interesting that the clustering of states using mode shares also independently clusters states with similar fatality risk. For instance, all the states within Clusters 1 and 3 have average or below average fatality rates. Cluster 2 has the highest median fatality risk and consists of 6 of the 10 states with the highest levels of fatality risk. This indicates a correlation between mode shares and fatality risk. The Pearson correlation between fatality rates and mode shares is –0.47 for walk, –0.23 for cycle, –0.26 for IPT, 0.5 for 2W, –0.19 for car, 0.35 for bus, and –0.16 for train.

### 5.2 Regression analysis

Table 3 presents mean and standard deviation of the posterior distributions of regression coefficients for the four models along with their significance based on 95% BCI. It is noteworthy that the direction of association (sign of coefficients) of different variables with the risk remains the same across all the four models. Thus, it is less likely that an omitted variable is leading to a biased result as far as the direction of association is concerned. The significance of some coefficients varies as more variables are added. Coefficients of cycle and 2W remain consistently significant across all the models, while the coefficients of walk, cycle, 2W and diesel, are significant across all models (2, 3 and 4) except 1.

**Table 3:** Regression model.

	Model 4 (Final)		Model 3		Model 2		Model 1	
	Mean	SD	Mean	SD	Mean	SD	Mean	SD
(Intercept)	−9.178^b^	1.030	−8.645^b^	0.937	−8.113	0.951	−10.214	0.592
ln⁡(bus)	0.066	0.109	0.073	0.108	0.112	0.112	0.114	0.125
ln⁡(IPT)	−0.234^b^	0.113	−0.198^a^	0.110	−0.157	0.114	−0.109	0.125
ln⁡(car)	0.263^a^	0.138	0.146	0.105	0.105	0.108	0.073	0.119
ln⁡(walk)	−0.355^b^	0.178	−0.281^a^	0.169	−0.434^b^	0.160	−0.275	0.156
ln⁡(cycle)	−0.200^b^	0.082	−0.160^b^	0.076	−0.152^a^	0.080	−0.220^b^	0.085
ln⁡(2W)	0.390^b^	0.169	0.238^a^	0.128	0.270^b^	0.135	0.462^b^	0.128
ln⁡(diesel)	0.264^a^	0.131	0.345^b^	0.117	0.331^b^	0.124		
ln(Length of NH (km))	−0.146^b^	0.068	−0.085^b^	0.041				
Proportion Urban population	−0.832	0.613						
Density	0.039	0.108						

a Significant at 90% BCI.

b Significant at 95% BCI.

The inclusion of diesel in model 2 has a significant effect on magnitude of coefficients of walk and 2W, and to a lesser extent, on the coefficient of IPT and cycle. The coefficient of walk increased while those of cycle and 2W reduced. In model 3, the addition of national highways results in the reduction of the magnitude of walk, almost negating the increase from the addition of diesel in model 2. The effects of proportion urban population and density in model 4 is insignificant, however, their addition to the model results in an increase in the magnitude of coefficient of car. The effect of car is also significant in model 4, while it is insignificant in the other three models. Model 4 is considered as the final model, and the rest of the discussion in the paper will be based on results of this model.

To summarise the results of the regression model, fatality risk is positively associated with distance travelled by 2W, car, and bus, and negatively associated with distance travelled by walk, cycle and IPT. The effect of bus is mixed because walking from bus also contributes to total walking distance and the latter has a negative effect on risk. IPT contributes to lower risk through vehicular distance (IPT has a negative coefficient) as well as through its contribution to walking distance. In addition, walking distance includes walking contributed by train. Therefore, all forms of PT indirectly contribute to reduced risk.

The direction of association of modes with fatality risk is consistent with the Pearson correlation between mode shares and risk as discussed in Section 5.1, except in case of car. The correlation in case of car is negative while its effect is positive in the regression model. This is likely because high share of car is correlated with low share of 2nW. Therefore, a negative correlation with risk is likely a reflection of low share of 2W. The regression model, on the other hand, estimates an independent effect of car.

The effects of NH and proportion urban population are negative, while density has a positive effect. The effect of national highways is most counterintuitive and is expected to be in the opposite direction. Since length of other road types has not been included, NH may be indicating the effect of overall road network. An increase in the density of road network may be an indicator of higher congestion. The effect of urban population indicates higher safety resulting from slower travel speed within urban areas, as opposed to the faster moving traffic on rural inter-city roads.

### 5.3 Sensitivity analysis

I conducted sensitivity analysis of assumption of access-egress distance for all PT trips longer than 1 km (see Appendix: Table A3). In the main analysis, distance of 1 km is assumed. For sensitivity, I assumed no distance and 1.5 km. For no distance assumed, the effect of bus becomes weaker and in case of 1.5 km it becomes stronger. This is expected because when no walking distance is assumed, bus coefficient represents a mix of positive effect of bus and a negative effect of walking, thus effectively reducing its positive effect. Also, the magnitude of walk coefficient reduces in case of no walking distance for PT. Again, the effect of including walking distance for PT is in the direction as expected. Without this distance, the effect will be mixed with the coefficient of bus. The effect of these assumptions on IPT coefficient was similar to that of bus.

## 6 Scenarios

To develop scenarios, I used average mode shares of the five clusters (Table 2) as the five baselines representing different travel patterns across the states in India. The scenarios have been modelled to illustrate safety effect of mode shift among three different pairs of modes—(A) 2W and car, (B) walk and 2W, and (C) cycle and 2W. The directions of mode shifts in the three pairs are 2W to car, walk to 2W, and cycle to 2W, respectively. The mode shift scenarios represent the gradual shift of road users from their current mode to a new mode as they acquire a new vehicle. For pedestrians and cyclists, the next affordable mode is 2W, and for 2W users, it is car. The shift to and from PT has not been included. Excluding intercept and other control variables, the model I use to develop scenarios has the form as shown in Eq. (6).

(6) $$f_{n}={walk}^{-0.36}{cycle}^{-0.2}{IPT}^{-0.23}{bus}^{0.07}{2W}^{0.39}{car}^{0.26}$$

The model exponents in Eq. (6) are the mean values of coefficients from the final model (model 4; Table 3). In the three scenarios, the mode shift was modelled in ten steps including the baseline. In each step, 0.5 percent points of mode share are shifted from one mode and added to another. This ensures that the total distance remains the same. For instance, for cluster 1, the baseline mode shares of car and 2W are 0.112 and 0.084, respectively. In the next step, the share of car is 0.117 (0.112 + 0.005) and that of 2W is 0.079 (0.084–0.005), and so on for further steps.

To estimate relative risk (RR) for each incremental step, number of fatalities ($f_{n}$) calculated for each step was divided by the fatalities calculated for the baseline. Therefore, in effect, the RR of baseline is one. Note that while the model was developed with total distance travelled, for the scenarios, I am using mode shares, which is effectively multiplying the original model with a constant.^1^ In case of Cluster 1 in scenario C, only 4 steps are modelled as the baseline mode share of cycle in the cluster is very low and approaches to zero by the fourth step.

**Fig. 3:**
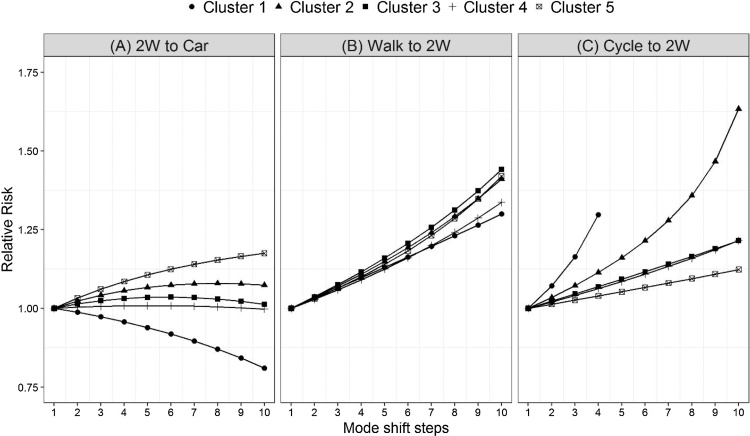
Relative risk for 5 clusters of Indian states resulting from incremental mode shift in three scenarios (Each step is 0.5% points shift from one mode to another).

Among the three scenarios (see Fig. 3), the growth rate in RR predicted for scenario B (Walk to 2W) and scenario C (Cycle to 2W) is much steeper than scenario A (2W to Car). RR is predicted to reach 1.3 to 1.5 in scenario B, 1.2 to 2.5 in scenario C, and 0.8 to 1.2 in scenario A. This is expected because in scenario A, mode shift is occurring among modes (2W and car) which are both associated with higher risk (positive exponent). On the other hand, in scenarios A and B, mode shift is occurring from low-risk modes with negative exponents (walk and cycle) to a high-risk mode with positive exponent (2W).

Unlike scenarios B and C, RR does not have a monotonic trend in scenario A. For instance, Cluster 1 experiences a decline in RR while Cluster 5 experiences a high growth rate. The other three clusters (2, 3 and 4), on the other hand, experience a slow rate of increase and have a slight U-shaped form. U-shape indicates a possibility of a critical point beyond which further mode shift from 2W to car results in higher safety. Thus, Cluster 1 consists of states which are already beyond that critical point, while all the states in the other four clusters will reach that point in the future. It is, therefore, worth investigating the travel patterns in Cluster 1 in relation to other clusters.

Among all the clusters, Cluster 1 has the highest mode share of car and the lowest share of 2W (see Table 2). The ratio of mode shares of cars to 2W in this cluster is 3.5 while this ratio ranges from 0.3 to 0.56 in other four clusters. In Cluster 5, which experiences highest growth in RR, mode share of 2W is the highest among all the clusters and ratio of car to 2W share is 0.3. Clearly, both Clusters 1 and 5 stand out in terms of their mode shares of 2W and car as well as relative ratios of the two modes. Cluster 1 represents a system with many more cars with a small number of 2W, and Cluster 5 represents the reverse. The same mode shift makes Cluster 1 safer while it makes Cluster 5 less safe. Interestingly, all the states in Cluster 1 are contiguous and are in the north-eastern part of the country.

## 7 Discussion

In this study an ecological regression model was developed at the state level to understand the relationship between road deaths and commute distance travelled by different modes in India. The relationship controlled for diesel consumed, length of national highways, percent population urban, and population density. The regression modelling was carried out using hierarchical Bayesian framework thus ensuring stable estimates with limited number of units of analysis. An additional benefit is that coefficients are reported in terms of distributions, and therefore, a subjective understanding of the coefficients can be made.

The regression model shows that walking, cycling, and IPT are associated with lower risk of road deaths in states. On the other hand, car, 2W, and bus are associated with higher risk. Thus, out of six on-road modes of transport, half are associated with lower risk, while the other half with higher risk. The two modes with the highest magnitude of the coefficients are walk and 2W, however, with opposite signs. The model thus represents the interaction of multiple modes on Indian roads and their competing effects towards risk. The coefficients imply that any mode shift from walking, cycling or IPT to any of the other three modes (2W, car and bus) is likely to result in higher number of road deaths.

An interesting finding is that IPT such as auto rickshaws or tuk-tuks are associated with lower risk of road deaths. This is in line with the safety hypotheses of auto rickshaws reported earlier (Mohan and Roy, 2003; Pandey et al., 2015). IPT vehicles have a small engine size of 300 cm^3^ which limits their speed to 50–60 km/h, compared to more than 1600 cm^3^ of an average car in India with much higher speed. Further, the weight of an auto rickshaw is one fifth of a small car. With smaller engine size and body weight, in case of a crash, auto rickshaw results in lower injuries to pedestrians and cyclists. At the same time, due to an enclosure, it provides safety to its occupants in case of a crash with a car or a heavier vehicle. Low capability of speeding also results in traffic calming of other motorised traffic. This finding has an important implication because IPT is the only motorised mode which is associated with higher safety. At the same time, it is a form of public transport and hence its growth is desirable from sustainable transport perspective.

The effect of bus is mixed with bus distance associated with higher risk of road deaths, and the walking distance (accompanied with bus trips) associated with lower risk. While a large proportion of passenger transportation in India is carried out through buses, the infrastructure is insufficient for the buses to operate safely. Very often buses in India share the road space with pedestrians and cyclists in the absence of dedicated infrastructure for the latter two road users (Tiwari et al., 1998). There are also bus design issues which result in higher number of deaths. Due to absence of automatic doors, passengers are injured from a fall during boarding or alighting. Due to high ground clearance of bus, in case of a crash, pedestrians and cyclists are crushed under the wheels of buses, which would be prevented with a low clearance (Kharola et al., 2010).

The effect of bus agrees with the results reported by Bhalla et al. (2007) who developed a risk-based injury model supported by empirical estimates of case fatality ratios. The authors also reported an increase in the number of deaths with a mode shift scenario in favour of buses. However, the number of deaths in bus scenario was much lower compared to those where mode shift occurred towards cars and 2W. The regression results in the present study also indicate that the coefficient of bus is positive (therefore the deaths will increase with higher bus share) but is much lower in magnitude than that of cars and 2W. Use of buses is desirable from the perspective of sustainable transport, and with low levels of vehicle ownership, a large section of society depends on buses for their daily travel. In this respect, road infrastructure needs to be designed and bus design needs to be modified to minimise the externalities of bus use.

Among the two private motorised modes, exponent of 2W is more than 1.5 times higher than that of cars. This indicates that for a given distance, 2W results in much higher risk than cars. Note that the dependent variable in the model is total road deaths. Thus, the coefficient for a vehicle type indicates risk of the vehicle occupants as well as those struck by it. 2W are hazardous for their riders as well as those struck by them such as pedestrians or cyclists. Cars, on the other hand, have much higher safety for their occupants, while highly hazardous to the colliding road users such as pedestrians, cyclists as well as 2W riders. This is likely why the coefficient for 2W is higher than cars. This is possibly why mode shift from 2W to car results in a U-shaped trend. As car share increases, beyond a certain mode share mix, the effect of lower risk of cars overcomes the higher risk of 2W, and the resulting effect is an overall reduction in number of deaths.

U-shape trend of fatalities observed in the 2W to car scenario has been empirically observed earlier in the road injury literature **(**Söderlund and Zwi,1995; Kopits and Cropper, 2003). According to these, road traffic injury burden in countries, often expressed as fatalities per capita, rises to a certain level of per capita income and then reduces with increasing income. Explaining this phenomenon, Bishai et al. (2006) concluded, among other factors, that the reduction is possibly because road users become safer as they shift from vulnerable modes of transport to cars with much higher safety. A similar finding was reported by Bhalla et al. (2007) using their risk-based model. In the scenarios involving mode shift to car, the authors found a U-shaped trend which they attributed to increasing safety of car occupants.

Scenarios of mode shift from walking and cycling to 2W present a worrying trend of fatalities if growth in 2W ownership continues to replace existing trips of walking and cycling. The fatalities according to the scenarios are predicted to increase at a steep rate. If past trends are any indicator, the steep growth of fatalities is not completely hypothetical. For instance, from 1996 to 2014, a period of less than 20 years, fatality rate increased by 3 times in Punjab state and 2 times in Chandigarh and Sikkim. Many other states also experienced high growth rate if not as dramatic as the three (Mohan et al., 2015).

With a high share of 2W in motorised fleet, India and many other south-Asian settings face a special challenge of road traffic injuries. For a low-income population, 2W present an option of owning a motorised mode with less than one-third the cost of buying a car, 3 times higher fuel efficiency (Goel et al., 2015), and lower parking space requirement. While 2W always have a higher risk than cars, these risks need to be minimised by strict enforcement of motorcycle helmets. The risks can be further reduced by the implementation of speed calming measures.

## 8 Strengths and limitations

The study has strengths as well as some limitations. This paper reports the use of census data to develop an injury prediction model accounting for exposure of all road users. For a setting like India with a complex mix of traffic modes, this study adds a significant understanding of how road death burden will evolve as travel patterns change in future. This is the first such study in India and the methods can be applied to model injuries at city or district level.

Given that it is an ecological study with large units of analysis, the results may be biased due to modifiable area unit problem. The number of fatalities within a state are an aggregate of urban and rural areas. In the rural areas, which include highways, both the type as well as the speed of traffic is different from the urban areas. On the highways, traffic is dominated by cars, buses and trucks, while within urban areas, traffic consists of many more pedestrians, cyclists, and 2W users. Though all types of road users can be seen in urban areas as well as on highways in India as the latter often pass through village settlements or towns. There is a possibility that distance travelled by cars, buses, and trucks will translate into different risk to its occupants as well as other road users in urban areas compared to rural areas.

The other limitation is the use of deaths as the measure of road injury burden, which represent only a small fraction of total crashes. It is also likely that while the deaths reduce in a scenario, the number of serious injuries may still rise.

## Figures and Tables

**Table A1:** Number of road deaths in each state over 2010–2012 period.

State	2010	2011	2012	Average
Arunachal Pradesh	139	126	136	134
Assam	2030	2342	2291	2221
Bihar	4693	5072	5056	4940
Chandigarh	138	136	114	129
Chhattisgarh	2888	3454	3167	3170
Dadra & Nagar Haveli	62	63	53	59
Daman & Diu	23	21	29	24
Goa	342	338	302	327
Gujarat	7384	8006	7855	7748
Haryana	5006	4681	4598	4762
Himachal Pradesh	1099	1083	1109	1097
Jammu & Kashmir	1029	1140	1426	1198
Jharkhand	2140	2053	2512	2235
Karnataka	9574	8958	9448	9327
Kerala	3950	4145	4286	4127
Madhya Pradesh	8539	8256	8506	8434
Maharashtra	14063	13680	13936	13893
Manipur	153	156	158	156
Meghalaya	184	229	213	209
Mizoram	82	81	77	80
Nagaland	44	36	56	45
Delhi	2170	2107	1866	2048
Puducherry	239	233	233	235
Punjab	2133	4897	4795	3942
Rajasthan	9163	9232	9528	9308
Sikkim	71	106	44	74
Tamil Nadu	15409	15422	16175	15669
Tripura	236	245	272	251
Uttar Pradesh	15099	14996	15109	15068
Uttarakhand	917	922	827	889
West Bengal	5470	5646	6222	5779
Odisha	4105	3797	3701	3868
Andhra Pradesh	15337	15158	14966	15154

**Table A2:** Average (Standard deviation) Trip Distance by mode in km for India and 33 states (Goel, 2018).

State	All Modes	Walk	Bicycle	Bus	Car	IPT	2W	Train
India	10.1 (16.5)	2.1 (2.3)	5.4 (7.8)	21.1 (26)	15.6 (28.4)	10 (16.2)	8.2 (14.2)	51.9 (62)
Andhra Pradesh	10.5 (16.8)	2.4 (2.4)	5.1 (7.4)	23.4 (27.7)	17.0 (26.8)	10 (15.7)	8.6 (15.1)	77.7 (77.8)
Arunachal Pradesh	5.0 (12.2)	1.8 (2.4)	4.1 (6.7)	21.9 (26.3)	12.3 (27.1)	8.9 (15.4)	7.3 (14.7)	23.6 (46.4)
Assam	7.3 (13.5)	1.5 (2)	4.2 (6.4)	33.8 (33.5)	14.6 (29)	8.1 (13.5)	7.2 (13.1)	55.5 (79.7)
Bihar	9.3 (15.6)	2.4 (2.5)	6.0 (8.5)	23.9 (34.9)	16.4 (31.2)	8.9 (14.4)	9.3 (16.3)	55.1 (76.4)
Chandigarh	7.0 (11.5)	1.9 (2.1)	5.7 (7.6)	17.3 (22.8)	9.3 (15.8)	7.9 (12.3)	6.5 (9.6)	36.6 (50.4)
Chhattisgarh	7.1 (12.9)	1.5 (1.9)	5.2 (7.5)	30.1 (41.2)	17.3 (32.6)	10.5 (16.6)	8.2 (14.4)	38.1 (57)
Dadra & Nagar Haveli	4.5 (9.1)	0.9 (1.7)	3.5 (5)	12.7 (17.8)	9.5 (18)	6.7 (10.2)	6.4 (11.3)	61.4 (85.1)
Daman & Diu	5.1 (11)	1.0 (1.5)	2.7 (3.8)	32.7 (32.4)	7.3 (13.9)	6.7 (10.6)	5.6 (10)	55.1 (79.8)
Goa	10.0 (16.6)	2.0 (2.3)	5.2 (7.5)	16.2 (21.8)	13.4 (25.2)	11.6 (19.6)	10.2 (17.4)	30 (45.5)
Gujarat	7.7 (13.4)	1.9 (2.2)	4.4 (6.3)	24.2 (28.3)	14.6 (26.6)	8.5 (13.5)	6.7 (11.4)	41.3 (57.8)
Haryana	12.9 (19.2)	2 (2.3)	5.4 (7.6)	38.9 (36.7)	17.7 (31.3)	10.2 (15.7)	8.4 (14.6)	53.3 (69.7)
Himachal Pradesh	9.1 (17.8)	2.1 (2.2)	5.4 (7.6)	19.2 (24.5)	13.1 (26)	13.5 (22.4)	9.1 (15.8)	40.9 (64.4)
Jammu & Kashmir	12.5 (23.2)	2.6 (2.5)	6.3 (8.7)	22.4 (26.9)	16.3 (28.7)	12.8 (20.2)	8.8 (14.4)	66.2 (88.6)
Jharkhand	7.9 (13.8)	2.2 (2.2)	6.3 (8.6)	29.5 (41.9)	13.8 (26.1)	9.4 (14.3)	7.5 (12.8)	55.7 (76.3)
Karnataka	10.6 (16.9)	2.2 (2.3)	5.7 (8.1)	18.4 (23.8)	15.7 (27.9)	12.6 (20.3)	8.6 (14.8)	64.8 (70.1)
Kerala	8.7 (15.8)	1.7 (2)	4.3 (6.1)	12.4 (17.6)	11.1 (20.8)	6.4 (11.4)	7.7 (12.4)	81.4 (99.4)
Madhya Pradesh	7.5 (13.4)	2.2 (2.3)	5.5 (7.8)	26.4 (29.8)	15.3 (29.5)	9 (14.6)	7.5 (13.4)	76.6 (77)
Maharashtra	10.2 (18.5)	2.2 (2.3)	5.0 (7.2)	17.0 (22.6)	14.5 (22.1)	10.3 (17.2)	8.8 (15.2)	38.7 (55.2)
Manipur	8.3 (16.2)	2.1 (2.6)	5.3 (7.7)	25.8 (35.2)	13.5 (26.3)	8 (13.2)	6.8 (11.5)	23.5 (43.7)
Meghalaya	8.5 (15)	1.8 (2.2)	4.7 (7.1)	36.0 (34.2)	14.8 (28.8)	6.9 (11.9)	9.6 (17.5)	26.5 (50.9)
Mizoram	4.3 (9.7)	0.9 (1.8)	3.4 (5.5)	8.8 (14.8)	10.3 (23.8)	4.3 (8.5)	4.5 (8.8)	24.8 (49.9)
Nagaland	4.6 (10.2)	1.2 (2)	3.2 (4.9)	13.4 (19.4)	10.4 (22.4)	6.7 (12.1)	5.7 (10.7)	21.6 (44.7)
Delhi	8.8 (14.2)	1.6 (1.9)	5.9 (8)	12.0 (15.7)	14.0 (18.6)	13.5 (21.6)	10.9 (17.4)	19 (23.8)
Odisha	9.5 (15.8)	2.2 (2.3)	6.2 (8.9)	33.6 (46)	19.7 (36.3)	12 (19.3)	9.9 (17.4)	60 (82.5)
Puducherry	8.8 (14.6)	1.9 (2.2)	4.8 (6.9)	18.1 (23.6)	14.9 (28.2)	9.1 (15.9)	7.1 (12)	38.1 (59.5)
Punjab	8.8 (14.9)	2.3 (2.5)	5.8 (8.2)	30.8 (32.5)	13.3 (24.5)	9.1 (14.6)	7 (12.2)	47.4 (69.7)
Rajasthan	10.4 (16.8)	1.9 (2)	5.1 (7.1)	32.8 (33.4)	15.8 (28.7)	9.4 (14.7)	7.9 (13.4)	75.2 (90.8)
Sikkim	5.6 (12.3)	1.7 (2.1)	7.0 (10.1)	14.6 (20.4)	12.6 (24.2)	9.9 (16.9)	9.7 (16.4)	36.6 (61.6)
Tamil Nadu	11.7 (18)	1.9 (2.2)	4.5 (6.6)	20.4 (25.5)	16.5 (29.4)	14.2 (23)	8 (13.6)	51.4 (61.6)
Tripura	6.1 (11.7)	1.7 (2)	3.9 (5.6)	24.3 (33.3)	16.9 (24.5)	7.2 (11)	7 (12.1)	18.9 (34.4)
Uttar Pradesh	10.6 (17)	2.3 (2.5)	6.6 (9.3)	36.1 (34.8)	16.3 (25.9)	10.9 (17.1)	9.6 (16.4)	85.2 (81.8)
Uttarakhand	8.3 (14.4)	1.9 (2.2)	5.5 (7.8)	29.5 (31.6)	16.6 (30.5)	9.2 (14.1)	7.3 (12.8)	38.5 (60.5)
West Bengal	12.1 (18.5)	2 (2.4)	5.0 (7.5)	21.4 (26.2)	11.7 (21.8)	10.5 (17.6)	8.5 (15)	41.2 (54.7)

**Table A3:** Sensitivity analysis of access distance of PT.

	Final model		Model assuming no distance for access-egress of PT		Model assuming access-egress distance of 1.5 km for PT	
	Mean	SD	Mean	SD	Mean	Mean
(Intercept)	−9.178^b^	1.030	−9.017^b^	1.065	−9.251	1.020
ln⁡(bus)	0.066	0.109	0.034	0.102	0.077	0.112
ln⁡(IPT)	−0.234^b^	0.113	−0.220^a^	0.116	−0.240^b^	0.113
ln⁡(car)	0.263^a^	0.138	0.248^a^	0.137	0.267^a^	0.139
ln⁡(walk)	−0.355^b^	0.178	-0.336^a^	0.171	−0.356^b^	0.180
ln⁡(cycle)	−0.200^b^	0.082	−0.187^b^	0.084	−0.205^b^	0.081
ln⁡(2W)	0.390^b^	0.169	0.373^b^	0.170	0.396^b^	0.169
ln⁡(diesel)	0.264^a^	0.131	0.269^b^	0.132	0.262^b^	0.131
ln(Length of NHs (km))	−0.146^b^	0.068	−0.139^b^	0.069	−0.149^b^	0.068
Proportion Urban population	−0.832	0.613	−0.855	0.619	−0.818	0.612
Density	0.039	0.108	0.044	0.108	0.038	0.108

a Significant at 90% BCI.

b Significant at 95% BCI.

## Footnotes

1 For example: ${\left(walk\;mode\;share\right)}^{-0.34}={\left(walk/total\;distance\right)}^{-0.34}={walk}^{-0.36}{total\;distance}^{0.34}$, where ${total\;distance}^{0.34}$ is a constant for a given state
